# Nutritional behaviors of women and men in Poland during confinement related to the SARS-CoV-2 epidemic

**DOI:** 10.1038/s41598-021-99561-w

**Published:** 2021-10-07

**Authors:** Izabela Bolesławska, Ewa Błaszczyk-Bębenek, Paweł Jagielski, Anna Jagielska, Juliusz Przysławski

**Affiliations:** 1 Department of Bromatology, Poznan University of Medical Sciences, Poznan, Poland; 2 Department of Nutrition and Drug Research, Institute of Public Health, Faculty of Health Sciences, Jagiellonian University Medical College, Krakow, Poland; 3 Department of Social Medicine and Public Health, Medical University of Warsaw, Warsaw, Poland

**Keywords:** Diseases, Health care, Risk factors

## Abstract

The influence of the confinement on the changes of eating behaviors in men and women in Poland and between groups were assessed. Results were obtained for 112 men and 200 women. An anonymous questionnaire available on-line from 29 April to 19 May 2020 was the research tool. It contained questions about the frequency of consumption "before" and "during" confinement. Additionally, anthropometric measurements were declared by the respondents. An increase in the number of meals and an improvement in their regularity were observed in both groups. However, the frequency of snacking also increased. During lockdown women consumed potatoes, sweets, canned meat and eggs and men consumed canned meat more frequently. Products consumed less frequently were: fast food, instant soups and energy drinks (women), and white bread and fast food (men). The frequency of alcohol consumption also increased during lockdown. Average body weight and BMI increased significantly during social isolation. Body weight increase was declared by almost half of women and 40% of men. During the blockade period caused by the COVID-19 pandemic, changes in the dietary behavior of the study group of women and men were found. The nature of these changes varied according to gender and the dietary parameters analyzed.

## Introduction

In Poland, the first case of severe acute respiratory syndrome SARS-CoV-2 was confirmed on March 4, 2020. By April 30, 2020, a total of 12,877 cases of COVID-19 were registered^1^. In November 2020, the incidence in Poland exceeded 20,000 cases per day^2^. By virtue of a decree of the Minister of Health, which was effective from March 14, 2020, on the declaration of an epidemic emergency in the territory of the Republic of Poland (Journal of Laws 2020, item 433, 441), a number of recommendations were implemented to reduce the risk of SARS-Cov-2 infection. Borders were closed, educational activities were banned, trade and the operation of restaurants and fast-food bars were restricted^1,3^. Remote working was mandated in many workplaces^4^. This resulted in many changes in daily life and routines such as reduced access to groceries, isolation and confinement at home and thus reduced work and physical activity^5,6^. Although from a public health perspective these actions were necessary to reduce the spread of COVID-19, their impact on health behaviors and lifestyles and long-term health consequences are difficult to determine^6,7^.

A multicenter study conducted in Asia, Africa, and Europe by Ammar et al^6^ found that isolation altered physical activity and eating behaviors in a health-threatening direction. Dietary intake and patterns (type of food, eating out of control, snacking between meals, number of main meals) were more unhealthy during lockdown. Among the negative changes in eating behaviors among U.S. adults during the COVID-19 pandemic were increases in unhealthy snacking, consumption of sweets, and sweetened beverages^5^. About one-third of U.S. families increased high-calorie snacks and desserts/sweets and nearly half increased non-perishable processed foods^8^. Sweet foods were also more frequently consumed among adolescents in Spain, Italy, Brazil, Colombia, and Chile^9^ and adults in Spain^10^. In contrast, the percentage of skipping breakfast remained constant during the COVID -19 pandemic in Kuwait compared to earlier, instead a snack or late night meal was more frequently consumed^11^. A worsening of the problem with excessive alcohol consumption was also frequently found^12^. In a study of adults in Italy by Di Renzo et al. it was shown that almost half of the respondents consumed comforting meals and were willing to increase their food intake to feel better^13^. Overeating behaviors have also been observed among the general Australian population^7^. Often unhealthy eating was accompanied by a decrease in physical activity^5,7,10,11^.

The consequences of social isolation were also manifested in positive changes in eating behaviors. Some studies observed decreases in alcohol binge drinking^6^, frequency of skipping breakfast^5^, decreases in eating fried foods^5^, meals eaten in restaurants^5,14^ and from fast food^5,11^ for an increase in eating fresh foods^8^ and a main meal freshly prepared^11^. The frequency of eating fruits^5,9^, legumes^9^ and vegetables^9^ also increased.

The imposed restrictions associated with the COVID-19 pandemic also changed some of the eating behaviors of Poles. A cross-sectional study by Sidor and Rzymski^15^ conducted in Poland during a nationwide quarantine showed an increase in food consumption and frequency of snacking, with changes in food consumption, snacking, and cooking during the quarantine not differentiated by age, sex, residence, education, or work status. Nearly 1/3 of the study group experienced weight gain and 1/5 experienced weight loss. It was also found that the blockade imposed on the infectious agent may affect eating behaviors and food habits especially among the most vulnerable groups including overweight and obese individuals. A study by Górnicka et al.^16^ confirmed increased food intake during COVID-19-induced lockdown in more than 30% of Polish subjects. Additionally, two opposing dietary patterns were observed: Prohealthy associated with increased intake of whole grain products, vegetables, fruits and water or Unhealthy in which increased intake of processed meat, fast food, confectionery and alcohol and sweets.

While short-term changes in dietary behavior should not cause serious health consequences, longer-term changes may result in the perpetuation of unhealthy dietary habits. The consequences are difficult to predict, but can be expected to adversely affect the development of diet-related diseases such as obesity, type II diabetes, and cardiovascular disease, which have been identified as potential risk factors for patients with COVID-19^17–23^.

The issue of higher mortality in men compared to women due to COVID-19 seems to be of interest. The reported mortality rates vary considerably between countries. However, almost all data show a nearly twofold increase in mortality in men compared to women^4,24–26^. The distribution of confirmed cases by sex in Poland did not differ from that in other European countries. By April 30, women accounted for 55.7% of all confirmed cases^3^, but men were much more likely to die^1^. Later in the epidemic, in China, Spain, Sweden and other countries^25,27–29^, both morbidity and mortality were higher in men^30^. In contrast, gastrointestinal and neurological manifestations of COVID-19 were more common in women^31^. The mechanisms responsible for the lower number of deaths in women are still unclear. One reason may be related to the higher prevalence of chronic lung disease, hypertension, and cardiovascular disease in men and their association with the severe course of COVID-19^27,32^. Gender-related body defense mechanisms that modulate the course of the disease are also not excluded^24,27^. Other factors, such as gender differences in risk behaviors related to cortisol levels (men are more likely to consume excessive amounts of alcohol or smoke cigarettes)^27,33^ may also influence the course of COVID-19. Most of the work published to date on COVID-19 has paid little attention to the impact of dietary factors on risk of infection and disease course by gender. And as numerous reports have shown, nutritional abnormalities in combination with numerous other risk factors may increase inflammation and thus morbidity and mortality^34,35^. This is especially true for men, who have poorer dietary habits and a higher prevalence of overweight and obesity than women. This calls for special attention to various strategies, including gender-targeted nutrition.

Given the higher morbidity and mortality and worse disease course in men compared with women associated with the COVID-19 epidemic, we set out to analyze the dietary behaviors of men and women during social isolation. The observed gender differences in susceptibility to COVID-19 underscore the need to understand the impact of various risk factors on morbidity and mortality and to tailor prevention according to gender.

## Materials and methods

To investigate the impact of the COVID-19 pandemic quarantine on the dietary habits of Poles, an anonymous online survey was conducted based on a self-designed questionnaire developed from the Dietary Habits and Nutrition Beliefs Questionnaire for people 15–65 years old^36^. The survey was uploaded and shared on Google's online survey platform. The link to the electronic survey was disseminated through various methods: invitation via email, sharing on social media sites Facebook, WhatsApp and Instagram. This provided an opportunity to reach a wide range of Respondents with pandemic safety, which was the recommended approach during the study period.

The retrospective research was conducted in Poland from 29 April to 19 May 2020, during social isolation in Poland. After initial verification, properly completed questionnaires of 112 men and 200 women were finally qualified for statistical analysis. The number of answers concerning anthropometric parameters varied. They were voluntary and anonymous. To measure body weight and waist circumference during lockdown, Respondents were given a measurement guide. Anthropometric results prior to social isolation were based on Respondents' memory.

All participants were informed about the purpose of the research and accepted the rules of data sharing and privacy before the survey. They also gave their informed consent to participate in the study. Persons under 18 years of age, with COVID-19, on a therapeutic or alternative diet and pregnant women were excluded. The completed questionnaire was sent to the survey platform of the Jagiellonian University and the final database was downloaded as a Microsoft Excel sheet.

The research was conducted in accordance with the principles of medical research ethics contained in the Helsinki Declaration. The consent of the Ethics Committee of the Medical University of Warsaw no. AKBE/122/2020 was obtained.

### Questionnaires

The questionnaire contained 96 questions prepared on the basis of the Dietary Habits and Nutrition Beliefs Questionnaire for people 15–65 years old, developed by the Behavioural Nutrition Team Committee of Human Nutrition, Polish Academy of Sciences^36^. The questions in Part I of the questionnaire asked about age, gender, education, occupation and place of residence^37^. Part II of the questionnaire included self-reported eating behaviors before and during the pandemic, including the following: number of meals eaten per day, meal regularity, frequency of snacking before and during social isolation.

The third part of the questionnaire concerned the evaluation of the frequency of consumption of 26 products and 7 beverages—with the answers: never, 1–3 times a month, once a week, several times a week, once a day, several times a day. The same questions were asked twice and referred to the period before and during social isolation.

The questionnaire was supplemented by questions on anthropometric data including body weight (kg), body height (cm) and waist circumference (cm)—before and during the pandemic. On the basis of the declared measurements the body mass index (BMI, kg/m^2^) was calculated for each participant at two time points: before and during social isolation. The BMI-based interpretation of the participants’ nutritional status conformed to WHO guidelines for adults^38^.

### Data analysis

Results for age and anthropometric data were presented as mean ± SD and median, other variables as percentage of total subjects. The Wilcoxon test or McNemar-Bowker test was used to check the differences for the variables before and during isolation. The Mann–Whitney U test used to check the differences for the variables between sex before and during isolation Data analysis was performed with PS IMAGO PRO 6 (IBM SPSS Statistics 26), assuming statistical significance level at < 0.05.

### Consent to participate

Informed consent was obtained from all individual participants included in the study.

## Results

### General characteristics of the examined group of men and women

Most of the men and women surveyed were from 36 to 45 years old, lived in the city, had higher education and a permanent job. Almost half of the surveyed group of women and men worked remotely from home during lockdown, and about 1/3 of respondents did not change the nature of their work. In the case of the vast majority of respondents of both genders the introduction of changes in the nature of work had little effect on the material situation, but in about 1/3 of the respondents it worsened. The general characteristics of men and women are presented in Table 1.

**Table 1:** General characteristics of the studied men and women (n = 312).

Analyzed parameter	Men (n = 112)	Women (n = 200)
Age (years) X ± SD (min–max)	42.1 ± 12.0 (18–72)	40.6 ± 13.69 (18–79)

	n (%)	n (%)
**Place of residence**		
City	92 (82.1)	151 (75.3)
Village	20 (17.9)	49 (24.7)
**Education**		
Primary + vocational	5 (4.50)	5 (2.50)
Secondary	21 (18.9)	44 (22.0)
Higher	86 (76.6)	151 (75.5)
**Job**		
No work	11 (9.90)	19 (9.50)
Parental leave	0 (0.00)	11 (5.50)
Odd job	6 (5.40)	13 (6.50)
Permanent work	91 (81.1)	136 (68.0)
Student	4 (3.60)	21 (10.5)
**Working during the lockdown**		
Work as before	37 (33.3)	30 (15.2)
Remote work from home	49 (44.1)	89 (45.2)
Childcare and care for a child under 8 years of age*	2 (1.80)	11 (5.60)
Leave	8 (7.20)	16 (8.10)
Not applicable	15 (13.4)	51 (25.9)
**Impact of the lockdown on the mateial situation**		
No change	68 (60.7)	138 (70.1)
Has improved	8 (7.20)	8 (4.10)
Has worsened	35 (31.5)	51 (25.9)

*n* number of participants, *%* percentage of respondents, *X* average, *SD* standard deviation.

*As a result of the Government's decision to close nurseries and pre-schools linked to the COVID-19 pandemic, parents of children under the age of 8 have been able to stay at home and claim childcare allowance from 12 March 2020.

### Change of anthropometric parameters before and during lockdown

The average male body weight before social isolation was 86.0 ± 15.6 kg and increased significantly during lockdown to 86.5 ± 16.3 kg (p < 0.0001). Similarly, body weight in women increased from 66.0 ± 12.2 kg from before social isolation to 67.0 ± 12.5 kg during lockdown (p = 0.0012). The Body Mass Index differed significantly in both periods both in men (26.5 ± 4.33 kg/m^2^ vs 26.7 ± 4.48 kg/m^2^, p < 0.0001) and in women (24.1 ± 0.70 kg/m^2^ vs 24.5 ± 4.21 kg/m^2^, p = 0.0010). However, the percentage of people in particular BMI ranges (normal body weight, overweight, obesity, underweight), depending on the analyzed period, showed no such differences (p > 0.05). The results are presented in Table 2.

**Table 2:** Anthropometric characteristics of the studied groups of men and women, including differences before and during SARS-CoV-2 (n = 312).

Analyzed parameter	Men X ± SD (min–max)			Women X ± SD (min–max)		
	Before	During	p	Before	During	p
Body weight (kg)	86.0 ± 15.6 (50.0–135)	86.5 ± 16.3 (50.0–137)	< 0.0001^a^	66.0 ± 12.2 (42.0–103)	67.0 ± 12.5 (43.0–106)	< 0.0012^a^
Body Mass Index (kg/m^2^)	26.5 ± 4.33 (17.3–42.6)	26.7 ± 4.48 (17.3–43.2)	< 0.0001^a^	24.1 ± 0.70 (16.4–36.5)	24.5 ± 4.21 (16.9–36.7)	< 0.0001^a^
Underweight (< 18.5 kg/m^2^), n (%)	1 (0.90)	1 (0.90)	0.7210^b^	5 (2.80)	6 (3.10)	0.8410^b^
Normal (18.5–24.9 kg/m^2^), n (%)	45 (41.7)	43 (39.4)		109 (60.2)	113 (57.3)	
Overweight (25.0–29.9 kg/m^2^), n (%)	42 (38.9)	45 (41.3)		52 (28.7)	56 (29.2)	
Obesity (≥ 30.0 kg/m^2^), n (%)	20 (18.5)	20 (18.3)		15 (8.30)	20 (10.4)	

*X* average, *SD* standard deviation, *n* number of participants.

^a^The Wilcoxon test.

^b^McNemar-Bowker test.

**Figure 1:**
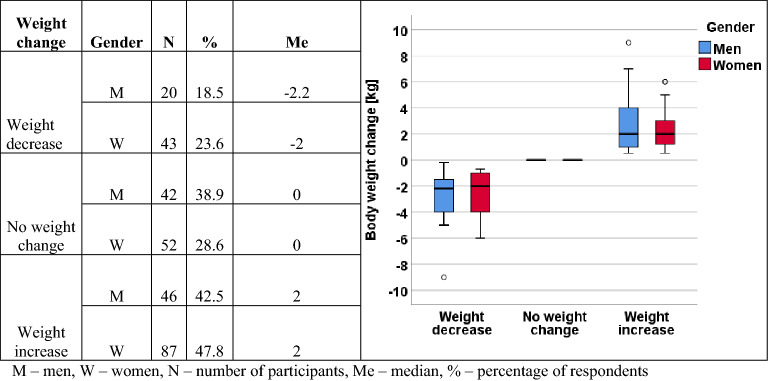
Changes in the body weight of men and women, including differences before and during lockdown. *M* men, *W* women, *N* number of participants, *Me* median, *%* percentage of respondents.

Weight changes comparing the previous period and lockdown are presented in Fig. 1. Weight gain was observed in 42.5% of men and 47.8% of women. Decreased body weight was noted in 18.5% of men and 23.6% of women, while only 38.9% of men and 28.6% of women did not report such changes.

### Nutritional habits of men and women before and during lockdown

**Figure 2:**
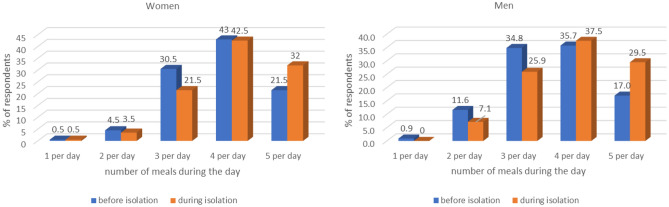
Number of meals consumed during the day.

During the period of social isolation the number of men consuming 5 meals a day almost doubled (p < 0.0001). A significantly higher percentage of women also reported eating 5 meals compared to the previous period (p < 0.0001) (Fig. 2).

**Figure 3:**
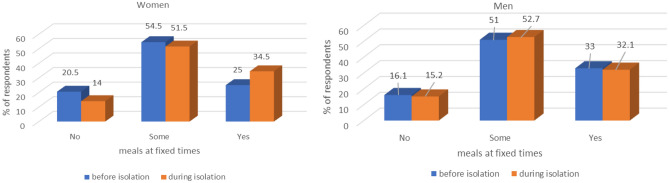
Meals eaten at fixed times by men and women before and during lockdown.

Over half of the respondents had regular meals before and during lockdown, but during lockdown the percentage of women who consumed all meals regularly increased significantly (p = 0.0127). The results are presented graphically in Fig. 3.

**Figure 4:**
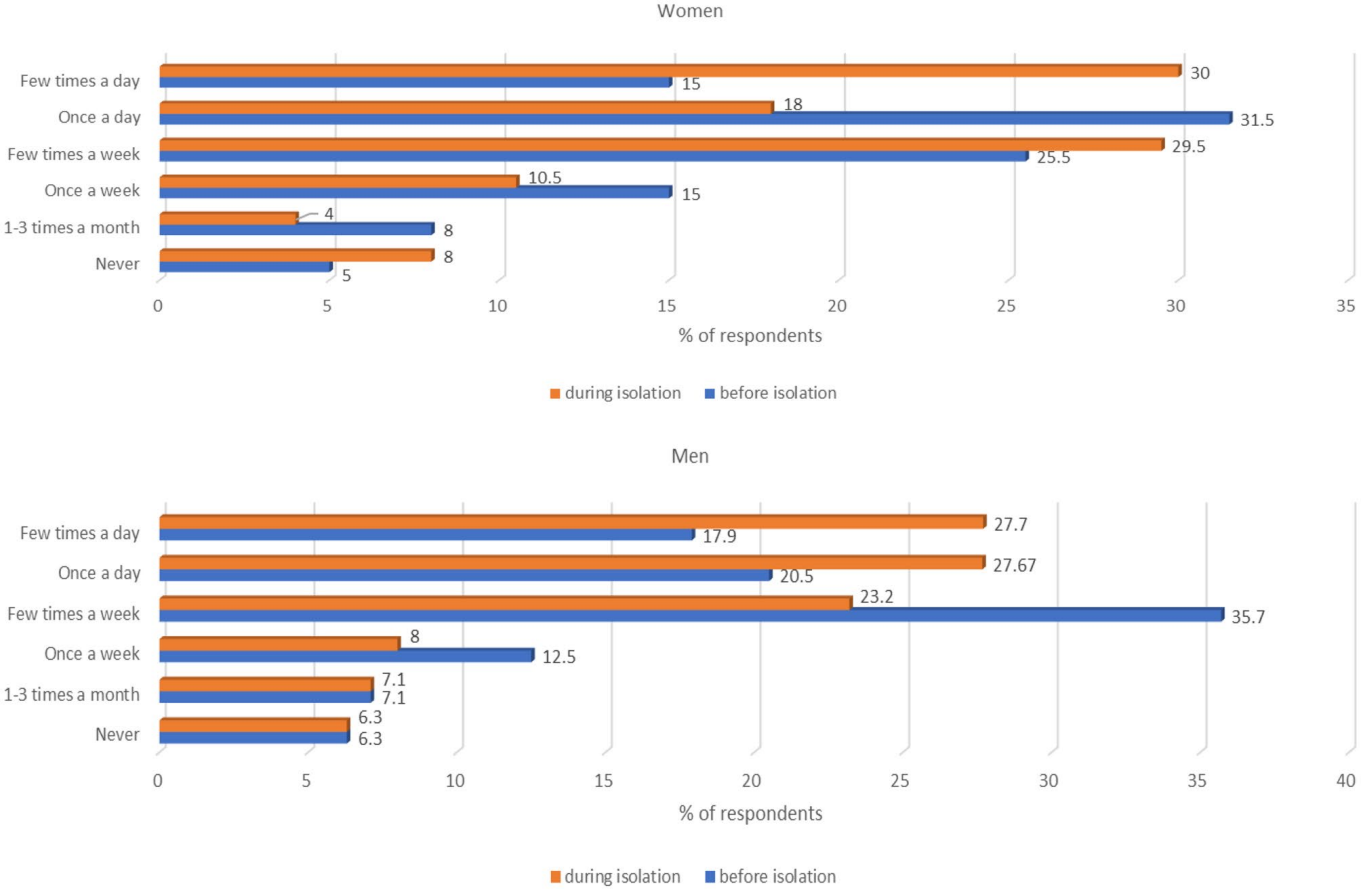
The frequency of snacking in a group of men and women before and during lockdown.

During social isolation, the frequency of snacking between meals also changed in both men and women. Before the introduction of quarantine, men usually snacked several times a week, while during lockdown they snacked once or several times a day (p < 0.0001). In women the percentage of those who snacked several times a day or several times a week doubled (p < 0.0001). The exact changes in the frequency of snacking in men and women are illustrated in Fig. 4.

Comparison of the frequency of consumption of selected products during the period preceding the introduction of restrictions related to the SARS-CoV-2 epidemic to the lockdown period in the study group of men showed a decrease in the frequency of consumption of white bread (p = 0.0372) and fast food (p = 0.0091), while an increase in the consumption of canned meat (p = 0.0091) during the lockdown period. For women, significant changes were observed during the lockdown compared to the pre-lockdown period, consisting of an increase in the frequency of consumption of potatoes excluding French fries and crisps (p = 0.0026), sweets (p = 0.0127), eggs (p = 0.0011) and canned meat (p = 0.0166), and a decrease in consumption of fast food (p = 0.0038) and instant and ready-made soups (p = 0.0327) (Table 3).

**Table 3:** Frequency consumption selected products, before and during lockdown (%).

Food product	Men (n = 112) % of respondents							p^a^ men/men B/D	Women (n = 200) % of respondents						p^a^ women/women B/D	p^b^ men/women B/D
	Time	Never	1–3 x/month	1 x/week	Few times /week	1 x/day	Few times a day		Never	1–3 x/ month	1 x/week	Few times /week	1 x/day	Few times a day		
Bakery products	B	6.30	2.70	13.4	25.0	23.2	29.5	**0.0372**	7.00	13.5	11.0	32.0	21.0	15.5	0.6663	**0.0020**
	D	4.50	6.30	19.6	24.1	18.8	26.8		7.00	10.0	14.5	33.5	18.0	17.0		0.0878
Wholemeal bread	B	19.6	22.3	15.2	18.8	14.3	9.80	0.7064	14.5	22.0	9.00	29.5	18.0	7.00	0.5625	0.2455
	D	22.3	13.4	16.1	31.3	9.80	7.10		19.5	13.0	16.0	27.5	17.5	6.50		0.3462
Butter	B	16.1	8.90	9.80	17.0	19.6	28.6	0.7942	11.0	9.00	7.00	27.0	22.0	24.0	0.7849	0.8473
	D	13.4	6.30	13.4	20.5	20.5	25.9		8.50	7.00	10.0	31.0	23.5	20.0		0.9530
Fermented milk	B	11.6	26.8	9.80	33.0	14.3	4.50	0.3958	9.00	15.5	14.5	41.0	17.5	2.50	0.7780	0.1190
	D	14.3	21.4	20.5	25.0	14.3	4.50		9.50	14.5	17.0	37.5	20.0	1.50		**0.0337**
Cheese	B	2.70	8.90	23.2	44.6	13.4	7.10	0.1748	6.50	16.5	19.5	45.5	10.5	1.50	0.6263	0.1680
	D	2.70	8.00	18.8	44.6	18.8	7.10		9.00	14.5	17.0	45.0	11.5	3.00		**0.0027**
Cold meats	B	5.40	12.5	18.8	37.5	15.2	10.7	0.9698	13.0	8.50	21.5	44.0	8.00	5.00	0.9211	**0.0278**
	D	5.40	7.10	21.4	41.1	19.6	5.40		11.0	12.5	20.0	42.5	8.50	5.50		**0.0115**
Eggs	B	4.50	8.90	28.6	48.2	7.10	2.70	0.4076	1.50	10.0	25.0	57.5	4.50	1.50	**0.0011**	0.6196
	D	1.80	8.90	27.7	52.7	7.10	1.80		1.50	6.50	22.0	57.5	11.0	1.50		0.1080
Potato	B	4.50	17.9	28.6	42.9	5.40	0.90	0.0649	5.00	19.0	26.0	45.0	4.00	1.00	**0.0026**	0.8939
	D	6.30	12.5	21.4	51.8	8.00	0.00		4.50	9.00	29.5	51.5	5.00	0.50		0.8190
Fruits	B	0.00	8.00	18.8	38.4	27.7	7.10	0.5951	1.00	4.50	8.00	33.0	33.0	20.5	0.9220	**0.0001**
	D	1.80	8.90	15.2	38.4	29.5	6.30		1.50	6.00	7.50	31.0	30.5	23.5		**0.0001**
Vegetables	B	0.90	3.60	14.3	40.2	30.4	10.7	0.4582	0.50	2.00	6.00	27.5	27.5	36.5	0.6747	**0.0000**
	D	1.80	6.30	12.5	41.1	25.0	13.4		0.50	2.50	4.50	29.0	29.0	34.5		**0.0000**
Fast food	B	21.4	53.6	15.2	9.80	0.00	0.00	**0.0091**	28.0	59.5	8.00	4.00	0.50	0.00	**0.0038**	**0.0149**
	D	31.3	51.8	10.7	5.40	0.90	0.00		47.5	36.5	12.0	3.50	0.50	0.00		**0.0215**
Fried products	B	3.60	24.1	28.6	42.0	1.80	0.00	0.5860	8.50	31.0	29.5	28.5	1.50	1.00	0.2971	**0.0128**
	D	5.40	24.1	28.6	39.3	2.70	0.00		11.5	22.5	29.5	35.0	1.50	0.00		0.1977
Sweets	B	5.40	20.5	17.0	41.1	13.4	2.70	0.7966	5.50	19.5	16.5	36.5	14.0	8.00	**0.0127**	0.4319
	D	6.30	21.4	17.9	32.1	15.2	7.10		8.00	13.0	14.0	35.0	16.5	13.5		0.0607
Instant soups	B	72.3	16.1	6.30	3.60	1.80	0.00	0.3892	76.0	16.0	4.50	2.00	1.00	0.50	**0.0327**	0.4137
	D	72.3	18.8	4.50	4.50	0.00	0.00		81.5	13.0	3.50	1.50	0.50	0.00		0.0570
Tinned meats	B	57.1	28.6	11.6	1.80	0.90	0.00	**0.0089**	82.0	15.5	1.50	0.50	0.50	0.00	**0.0166**	**0.0000**
	D	51.8	25.9	16.1	5.40	0.90	0.00		77.5	15.5	5.50	1.00	0.50	0.00		**0.0000**
Energy drinks	B	69.6	21.4	1.80	5.40	0.90	0.90	0.3539	83.5	11.0	1.50	3.50	0.50	0.00	**0.0240**	**0.0050**
	D	75.0	14.3	6.30	2.70	0.90	0.90		91.0	5.00	1.50	1.50	0.50	0.50		**0.0002**
Alcohol	B	11.6	41.1	21.4	16.1	7.10	2.70	**0.0301**	27.5	36.0	18.5	16.0	2.00	0.00	**0.0442**	**0.0021**
	D	14.3	28.6	24.1	20.5	9.80	2.70		32.0	24.5	17.5	23.0	3.00	0.00		**0.0015**

Bold indicates statistically significant differences (p < 0.05).

Women during mandatory quarantine were more likely to consume fermented milk drinks (p = 0.0337) and sweets (p = 0.0607) than men, while men consumed hard cheeses (p = 0.0027).

Before the social isolation associated with the COVID-19 pandemic, white bread and fried products were more frequently consumed by men than women (p = 0.0020 and p = 0.0128). In both analyzed periods, both before and during the lockdown, men were also more likely to declare consumption of cold cuts, sausages or Vienna sausages (p = 0.0278 before the lockdown and p = 0.0115 during the lockdown) and fast food (p = 0, 0149 before lockdown and p = 0.0215 during lockdown) and canned meat (p < 0.000 before lockdown and p < 0.000 during lockdown), while women were more likely to consume vegetables (p < 0.000 before lockdown and p = 0.000 during lockdown) and fruits (p < 0.000 before lockdown and p < 0.000 during lockdown) (Table 3).

The products consumed most often (several times a day) in the group of men, regardless of the analyzed period (before and during lockdown) were white bread, e.g. wheat, rye, mixed wheat-rye and rolls (29.5% before lockdown vs. 26.8% during lockdown; p = 0.0372) and butter, used as an additive to bread or food, e.g. for frying/baking. It was used several times a day by 28.6% of men before confinement and 25.9% during it (p = 0.7942). Vegetables (10.7% before lockdown vs. 13.4% during lockdown, p = 0.4582) and cold cuts, sausages or Vienna sausages (10.7% before lockdown vs. 5.40% during lockdown, p = 0.9698) were also frequently chosen by men.

The most popular product in women was vegetables consumed several times a day by 36.5% of respondents before lockdown and 34.5% during lockdown (p = 0.6747). Fruit (20.5% before lockdown vs 23.5% during lockdown, p = 0.9220) and butter (before lockdown 24.0% vs 20.0% during lockdown, p = 0.7849) were less popular. The details are presented in Table 3.

Significant differences in the group of men and women were related to the increase in the frequency of alcohol consumption during social isolation (p = 0.0301 and p = 0.0442). The consumption of energy drinks among women also decreased significantly compared to the previous period (p = 0.0240). Comparing the frequency of the consumption of selected drinks according to gender in both periods (before and during lockdown) a gender differentiation was found in both periods (p < 0.05). The results are summarized in Table 3.

Significant differences between men and women included an increase in the frequency of alcohol consumption during social isolation compared to before lockdown (p = 0.0301 and p = 0.0442). There was also a significant decrease in the consumption of energy drinks during social isolation among women compared to the pre-lockdown period (p = 0.0240). Comparing the frequency of consumption of selected beverages according to gender in both periods (before and during lockdown), there was variation by gender in both analyzed periods (before lockdown/ during lockdown) (p < 0.05). A summary of the results is presented in Table 3.

### Limitations

In the present study, we aimed to illustrate as accurately as possible potential changes in the frequency of food intake in Polish men and women before and during lockdown. Despite the large amount of data, we are aware of some limitations of the study that should be taken into account when evaluating the results.

Survey research is susceptible to the inherent limitations of self-reported outcomes. The survey was conducted using the Internet, without face-to-face contact, which was recommended during the COVID-19 pandemic but, especially for anthropometric measurements, could have caused measurement error. In order to capture the rapidly changing environment, it was not possible to collect results from the period before the outbreak of the COVID-19 pandemic and from the duration of the pandemic in the same way. Results of anthropometric measurements during the COVID-19 pandemic were obtained from self-reported measurements taken by study participants, while results from the period prior to the pandemic were obtained retrospectively, based on participants' memory.

The study also included retrospective data on eating behaviors and food intake fractions from the time before the blockade, which may have influenced the eating behaviors presented.

Also the fact that the electronic questionnaire was more frequently completed by respondents with higher education and from larger cities, probably due to greater availability of computers and better quality of Internet connections, limited the representativeness of the group in relation to the general Polish population, but given the recommendations for limited contact it was preferred.

The questionnaire itself was quite long as it contained almost 100 questions covering two time periods. The possibility of stopping the survey at any time and/or not answering all questions resulted in not all Respondents providing complete answers. Of all the surveys administered, we accepted for analysis only those that had fully completed sections on general data and dietary behavior. Unfortunately, this caused the size of the study group to decrease significantly. Limitations of the study also include the fact that a large percentage of the respondents did not provide answers regarding anthropometric parameters or provided answers only regarding the time of the COVID-19 pandemic.

The form of survey we adopted, despite some shortcomings, is certainly the preferred solution during the COVID-19 pandemic.

### Discussion

The spread of COVID-19 resulted in numerous changes to the food chain in Poland, especially in terms of food purchase and consumption^39^. Nearly half of Poles were reluctant to stay in larger human concentrations^15^. Grocery stores became places of increased risk of infection, so Poles bought products to stock up^40^—mainly dry food, necessities, and took advantage of Internet shopping^41^.

Reduced ability to purchase food, greater availability of stockpiled products and more time spent at home contributed to changes in food consumption during the COVID-19 pandemic. Gender differences in food consumption during the COVID-19 pandemic were observed by Ruiz-Roso et al. in adults with type 2 diabetes mellitus (T2DM)^42^ and Sidor and Rzymski^15^ in the adult Polish population. The results obtained in our study also showed that changes in the structure of purchased products differently affected the eating behavior of the analyzed group of women and men. During the blockade, women were more likely to consume potatoes, sweets, tinned meat and eggs, whereas men consumed tinned meat. In contrast, the products consumed less frequently were fast food, instant soups and energy drinks (women) and white bread and fast food (men). The frequency of alcohol consumption also increased during the blockade. Reduced physical activity as a result of the COVID-19 pandemic restrictions translated into changes in anthropometric parameters.

Compared with previous studies conducted in Poland^15^, our study included a large group of men in addition to women. In addition, unlike the young people who participated in previous studies (mean age: 27.7 ± 9.0 years), here predominantly adults aged 36 to 45 years were surveyed. Special emphasis was also placed on observing gender differences in eating behaviors during the COVID-19 pandemic, but the study group was not analyzed as a whole, as it was the case in the studies by Błaszczyk-Bębenek et al.^37^ and Górnicka et al.^16^.

In our study, responses were obtained mainly from college-educated individuals from large cities, most of whom were working from home because of the introduction of COVID-19^4^ pandemic restrictions (Table 1). In about one-third of the respondents, the nature of work did not change. Also in other surveys conducted during the COVID-19 pandemic, responses were obtained from individuals who had access to a computer^5,43^ and were predominantly college-educated^6,44^.

A review of studies conducted by Babicz-Zielińska and Jeżewska-Zychowicz confirmed the significant influence of economic factors, especially income, on consumers' diets^45^.

The COVID-19 pandemic has caused a global crisis that has affected the socio-economic situation of families^46,47^. As of mid-March 2020, more than 30 million Americans (about 20% of the workforce) have filed for unemployment benefits^48^. In Pakistan, as a consequence of the COVID-19 pandemic, poverty levels are projected to increase by 33.7%^49^. Similar concerns apply to other countries around the world^47,50^. As evidenced by the statements of the Respondents who took part in our survey, the economic situation among Poles looks similar. In the case of the vast majority of respondents of both genders, changes in the nature of work had little effect on the material situation, but in the case of about 1/3 of respondents it worsened. These observations are confirmed in the report from research conducted by the Federation of Consumers^51^ on the finances of Poles during COVID-19. The report shows that over 60% of Poles do not feel the negative impact of the pandemic on their finances, while 16% limit their spending.

The mean duration of isolation among the men and women participating in the study was 51 days. This raises concern primarily in terms of weight gain or loss, as changes in food intake were accompanied by decreased physical activity and stress associated with adaptation to a new situation, such as national quarantine^6,55^. Physical inactivity and poor mental health caused by unexpected unpleasant events are among the most important risk factors for the incidence of serious diseases. Prolonged quarantine may represent a significant dietary risk, as both excessive weight gain and weight loss are associated with a more severe clinical course of SARS-CoV-2 and risk of death^15,52,53^. The present study demonstrated a significant increase in body weight and BMI during social isolation in study groups of Polish men and women. An increase in body weight was observed in almost half of the women and more than 40% of the men studied. A decrease in body weight was observed in 1/5 of the studied population. The study by Sidor and Rzymski^15^ conducted among young Poles in quarantine (mean age: 27.7 ± 9.0 years), mainly women (95.1%), showed that an increase in body weight was observed in almost 30% of them and a decrease in body weight in 19%. Changes in body weight in men and women during hospitalization were also found by He et al.^54^.

The introduction of restrictions on leaving residence during the COVID-19 pandemic contributed to various changes in dietary habits. Isolation was found to have a beneficial effect on meal frequency among both men and women. Flanagan et al. in a study conducted in the United States observed a reduction in breakfast skipping^5^. In Italy, on the other hand, more than half of the people surveyed did not change the number of daily meals, but almost ¼ of the respondents introduced an extra meal^13^.

Meal skipping is the omission or failure to eat one or more of the traditional main meals (breakfast, lunch, or dinner) during the day^56^. Regular skipping of meals, especially breakfast, is associated with poorer diet quality, increased risk of obesity, insulin resistance, and cardio-metabolic disease^57^. The study found that almost twice as many men and 10% more women ate meals 5 times a day compared with the previous period. A similar increase in the number of meals consumed was observed by Sidor and Rzymski and Ammar et al.^6,15^. Eating 5 meals allowing flexibility in the order and time of consumption of individual meals is common in the typical diet of Poles. In addition to the basic three meals, Poles usually eat a second breakfast consisting of sandwiches and tea^58,59^ while an afternoon snack usually consists of a sweet or salty snack. It has also been shown that the time spent at home during social isolation caused by COVID-19 epidemic had a positive effect on the number of meals eaten at a fixed time by women. Unfortunately, this was not the case for men.

Long-term quarantine may lead to frequent snacking due to boredom and stress^60^. Unfortunately, the frequency of snacking increased significantly in the Polish group during isolation (p < 0.0001). The percentage of women who snacked several times a day during isolation doubled compared to the previous period. The number of men who snacked once or several times a day also increased (10% and 7%, respectively). Increased frequency of snacking during the COVID-19 pandemic was also observed by Sidor and Rrzymski in more than half of the Polish respondents^15^, Ammar et al. in respondents from West Asia (36%), North Africa (40%), Europe (21%), and other countries (3%)^6^, Ruiz-Roso et al. in a cohort of adult T2DM patients from Madrid, Spain^42^, and Ruiz-Roso et al. in adolescents from Italy, Spain, Chile, Colombia, and Brazil^9^.

An important aspect of the study was the analysis of changes in the frequency of consumption of selected products according to the period studied and gender (Table 3). Here, both favorable and unfavorable changes in diet quality associated with the introduction of social isolation were demonstrated.

According to WHO recommendations, eating at home reduces the frequency of contact with other people and the likelihood of exposure to COVID-19^61^. In addition, eating out tends to be associated with higher intakes of energy, fat, sugar, salt, and low-quality foods compared with consumption at home^45,62^. An extremely positive change, also from a nutritional perspective, was a decrease in the frequency of fast food consumption regardless of gender during the COVID-19 pandemic compared with an earlier period. Ruiz-Roso et al. also observed a decrease in consumption of such foods during the isolation associated with the COVID-19 pandemic^9^.

A favorable trend was also associated with a decrease in white bread consumption during SARS-CoV-2-related isolation in men, but it remained the dominant food. White bread may contribute to the manifestation of chronic inflammation and autoimmune diseases^63^. Whole grain bread with anti-inflammatory effects is more beneficial in this aspect^64,65^. Meanwhile, the frequency of wholemeal bread consumption did not change during social isolation.

The decrease in the frequency of consumption of instant soups may indicate that women spent more time preparing meals in the traditional way. This is also confirmed by the higher consumption of potatoes during social isolation compared to the previous period. According to the literature, cooking at home, when done according to proper nutrition, can reduce the incidence of chronic diseases^66^ that cause increased mortality from COVID-19^25^. Sidor and Roman^15^ and Ruiz-Roso et al^9,42^ also reported more frequent cooking in a significant percentage of subjects during lockdown.

The altered pattern of consumption during quarantine resulted in an increase in the frequency of canned meat consumption by both men and women. This is of concern given the proven contribution of processed meat to the development of cardiovascular disease and cancer^67,68^. In addition, higher consumption of ultra-processed foods is strongly associated with a higher risk of multiple indicators of obesity^69^. An increase in processed meat consumption during quarantine was not observed in a study conducted among adolescents by Ruiz-Roso et al.^9^. This may be due to the characteristic high consumption of meat and meat products among Poles, especially men^70^.

Of great concern is the almost 10% increase in the percentage of women consuming sweets at least once a day during social isolation compared to before. It is now recommended to limit sugar and sweets as they can significantly increase fat accumulation and contribute to carbohydrate disturbances^71^. In Sidor and Rzymski study, 1/3 of the subjects admitted to consuming sweets daily during lockdown^15^. Increased intake of sweets and sweetened beverages was also observed during social isolation in the United States^5,8^. Sweetened foods were also more frequently consumed among adolescents from Spain, Italy, Brazil, Colombia and Chile^9^ and adults in Spain^10^.

The frequency of fruit and vegetable consumption was unfavorable in the male group. Although it did not decrease during the COVID-19 pandemic isolation compared with the pre-pandemic period, the frequency of their consumption in both periods analyzed was lower than in the female group. Many of the compounds in vegetables have positive effects on the cardiovascular system, prevention of diabetes, cancer, and reduction of anxiety and depressive symptoms^72,76^, which is extremely important during the COVID-19 pandemic, as these diseases may increase the risk of a more severe COVID-19.

Consumption of vegetables containing micronutrients and bioactive compounds is part of a non-pharmacological intervention to maintain normal immune system function. Therefore, increasing vegetable consumption during the COVID-19 pandemic seems highly advisable. Before and during isolation, more than 30% of the female study population reported consuming vegetables several times a day as recommended by WHO^73^ (the most commonly consumed products among women). In men, this percentage was just over 10% in both periods. This confirms previous observations that women presented more favorable eating behaviors than men^74^. There was also no change in the frequency of fruit portion consumption in the male group during lockdown compared to the pre-COVID-19 pandemic period. Similarly low frequency of fruit and vegetable consumption in Polish men was observed by Sidor and Rzymski^15^. Slightly different results were obtained by Ruiz-Roso et al. where an increase in fruit and vegetable intake during isolation was shown in both girls and boys^9^. WHO recommends fruits and vegetables as the best foods during quarantine or prolonged home confinement^75^.

Changes in beverage intake were only related to a decrease in the frequency of energy drink servings in the female group during social isolation compared to the previous period. Similarly, Ruiz-Roso et al. reported no significant changes in beverage consumption during COVID-19-related isolation^9^.

An increase in the frequency of alcohol consumption during quarantine compared to the previous period was declared by the vast majority of respondents, unlike in the study by Sidor and Rzymski^15^. A survey of Poles from April 10 to 22, 2020 found that nearly 30% of respondents consumed risky amounts of alcohol, and 14% of respondents had increased their alcohol consumption since the start of the pandemic^77^. Chronic alcohol exposure has complex and adverse effects on the body^78^, including innate and acquired immune mechanisms, and is known to increase susceptibility to viral infections^15^. Its excessive consumption may increase susceptibility to COVID-19 infection and exacerbate its clinical course^79^.

On the basis of the conducted studies, both favorable and unfavorable changes in the dietary habits of Polish women and men were found, with several gender-related differences. An increase in the number of meals observed among both men and women, as well as their regularity—especially among women, can be classified as health-promoting changes. On the other hand, increased frequency of snacking during the day among men and women may result in weight gain and lead to consequent diseases such as obesity, type II diabetes, cardiovascular diseases or various forms of cancer. In addition, the reduction in physical activity caused by the introduced restrictions most likely resulted in weight gain in almost half of the women and more than 40% of the men. Interestingly, about 20% of the women and men showed a decrease in body weight. An additional trigger for the weight changes (up or down) may have been the stress associated with the COVID-19 pandemic. No less, these changes occurred in a short period of time, raising concerns about worsening this trend if the lockdown is prolonged or repeated. The consequences are difficult to predict and may not be noticeable, but can also be expected to adversely affect the development of diet-related diseases such as obesity, type II diabetes, and cardiovascular disease, which have been identified as potential risk factors for COVID-19 patients^17–23^.

Similarly, the observed changes in the dietary behaviors of men and women in Poland if short-term then should not cause serious health consequences. However, their longer duration may result in the perpetuation of inappropriate eating habits. A review of studies conducted by Martinez-Ferran^55^ as the main metabolic consequences of the impact of several weeks of physical activity restriction combined with modified dietary habits on health identified an increase in insulin resistance, total and visceral adipose tissue and inflammatory cytokines and thus an increased risk of metabolic syndrome, which increases the risk of many chronic diseases^9,55,80^. In addition, previous pandemics have shown that the consequences of a pandemic can last longer and have a greater impact on lifestyle and mental health than the pandemic itself^81^. In order to avoid adverse health consequences, it seems necessary to pay attention to gender-specific eating behaviors if the situation associated with the introduction of social isolation were to recur.

## Conclusions

In the wake of the SARS-CoV-2 pandemic, Poles' dietary behaviors regarding the regularity and quantity of meals consumed have changed to be more health-promoting. The average body weight and BMI increased in half of the women surveyed and over 40% of men. Both men and women saw their meal intake increase and their regularity improve. However, the frequency of snacking between meals increased.

The frequency of food consumed did not clearly indicate improved nutritional awareness among women and men. The frequency of intake was shown to vary by gender and study period. There was increase in the prevalence of sweet consumption among women and canned food and alcohol consumption in both study groups, which if sustained over time could lead to serious health consequences.

This demonstrates the need to promote the role of proper nutrition in both male and female groups. Favorable trends were observed in the reduction of consumption of fast-food, powdered and ready-made soups and energy drinks in the diet of women and white bread and fast food among men.

## Data Availability

All authors declare that all data and materials as well as application support comply with field standards. Data supporting the results of this study are available from the authors.
